# The Antioxidant Properties of Selected Varieties of Pumpkin Fortified with Iodine in the Form of Potassium Iodide and Potassium Iodate

**DOI:** 10.3390/foods12142792

**Published:** 2023-07-23

**Authors:** Agata Zaremba, Marzanna Hęś, Anna Jędrusek-Golińska, Monika Przeor, Krystyna Szymandera-Buszka

**Affiliations:** Department of Gastronomy Science and Functional Foods, Faculty of Food Science and Nutrition, Poznań University of Life Sciences, Wojska Polskiego 31, 60-624 Poznań, Poland; agata.zaremba@up.poznan.pl (A.Z.); marzanna.hes@up.poznan.pl (M.H.); anna.jedrusek-golinska@up.poznan.pl (A.J.-G.); monika.przeor@up.poznan.pl (M.P.)

**Keywords:** fortification, pumpkin, iodine carriers, oxidative stability, vegetables

## Abstract

This study aimed to investigate the use of selected pumpkin varieties as carriers of potassium iodide (KI) and potassium iodate (KIO_3_) at different concentrations (2.3, 0.23, and 0.023 mg/100 g). It was hypothesized that the concentrations and form of iodine fortification in pumpkins affect the antioxidant activity of pumpkins. The results showed a high recovery of the introduced iodine in all pumpkin varieties after drying, as well as high iodine stability during storage, especially for KIO_3_. However, statistical analysis confirmed a relationship between the forms and concentration of iodine and the ABTS cation radical and the DPPH radical test results. In the systems with iodine concentration at 0.023 and 0.23 mg/100 g, the antioxidant activity did not change. However, for all pumpkin varieties fortified with a KIO_3_ concentration at 3.9 mg/100 g (2.3 mg/100 g of iodine), a statistically significant decrease in free-radical scavenging was confirmed. Therefore, for maximum effectiveness in pumpkin’s free-radical scavenging indices, it is suggested to introduce iodine in the form of KI and KIO_3_, but in controlled concentrations. However, KIO_3_ should be added at a maximum amount of 0.39 mg/100 g.

## 1. Introduction

Pumpkin belongs to the *Cucurbitaceae* family and is widely used in cuisines worldwide. For example, pumpkin (Cucurbita moschata Duch ex Poir) is one of Mexico’s most significant vegetable crops. It is commonly cultivated in South Asia, Africa, India, Latin America, and the United States [1,2]. This vegetable is frequently consumed in Mediterranean populations and is often cooked in many ways [3]. In industry, it is used to make baby food, juices, and marinades [4]. The use of pumpkin as a natural coloring agent in pasta and flour mixes has also been found [5,6]. Due to the lack of dominant sensory characteristics, pumpkin flesh is an excellent addition to many other products [7,8]. The industrial food applications of pumpkins also include meat products (beef meatballs, chicken burgers, and low-fat meatballs), grain products (bread, cake, biscuits, cookies, muffins, and pasta), beverages (juice, pineapple juice, and smoothies), and dairy (yoghurt and ice cream) [1,8,9,10]. It can also be incorporated into foods such as candies and rice cakes, as well as the traditional Indonesian cake ‘Bingka’ [11,12]. Enriching food products with pumpkin can be considered a strategy to increase the consumption of vegetables and the bioactive ingredients present in them without significantly changing eating habits of the population [12,13,14,15]. This is especially true of starch products, e.g., pasta and noodles. Therefore, in many studies, attempts have been made to improve the nutritional properties of these products through enrichment with vegetables, including pumpkin [8,16,17]. A significant advantage of pumpkin fruit is the low level of absorption of pollutants from the soil, thanks to which its flesh is characterized by a much lower level of heavy metals than in other vegetables [18]. Moreover, pumpkin has experienced increased interest in recent years because of its nutritional and health-protective effects. Pumpkin pulp is a good source of potassium, calcium, and carotene [5,19,20]. Pumpkin fiber is characterized by a high content of pectins, which lowers starch digestion and reduces the risk of diabetes [21]. Moreover, it contains crucial active substances such as avenasterol, spinasterol, sitosterol, and stigmasterol, as well as bioactive substances such as triterpenoids, sesquiterpenoids, carotenoids, tocopherols, and polyphenols [12]. For example, improved lipid profile and lowered blood pressure have been shown after eating the anatomical parts of pumpkin [10]. Moreover, the antioxidant properties of pumpkin extracts have also been confirmed [5,22,23]. 

Other results suggested the addition of pumpkin pulp to dairy products, e.g., ice cream [24]. In these studies, it was confirmed that the addition of pumpkin pulp to ice cream increases the nutritional value of this product. Following the addition of pumpkin pulp at the level of 10–20% to ice cream, the vitamin C content in these products was significantly increased. The antioxidant activity of these products was also high. The DPPH radical-scavenging activity of ice cream with pumpkin pulp significantly increased with increasing pumpkin substitution level (from 10% to 20%).

Food fortification is the most commonly used strategy to alleviate human nutrients deficiencies [25]. Previous studies also showed that pumpkins could be a suitable matrix for many nutrients, e.g., β-carotene (vitamin-A), thiamine, and calcium [1,26,27]. The study of de Escalada Pla also found that pumpkin flesh is an adequate raw material for the development of functional food fortified with iron [13]. Our preliminary research showed that pumpkin could also serve as raw material for iodine fortification [28].

Approximately 30% of the world’s population remains at risk of iodine deficiency [29]. From a public health perspective, pregnant women, fetuses, neonates, and infants are the most vulnerable groups because of the irreversible effects of iodine deficiency disorders (IDD) [30]. 

Iodine deficiency may be associated with exclusion from the diet of primary sources of iodine. Avoiding the consumption of animal-sourced food (vegan diet) may also be related to hypothyroidism. Vegans eliminate the primary sources of iodine from their diet, such as dairy products and fish, which makes them a group at particular risk of deficiency of this element [25,31,32,33,34,35,36]. Programs for food fortification with iodine are carried out in many countries worldwide to minimize the risk of this element’s deficiency in the diet [36,37]. One of the most common fortification strategies is salt iodization. However, in 2006, the World Health Organization recommended limiting salt intake to 5 g/day, as it is a risk factor for atherosclerosis and hypertension [38]. Consequently, the iodine supply from this source can be limited [32,39,40,41]. There is a danger of returning to the state from before the fortification of salt with iodine. Therefore, there is an urgent need to introduce new matrices for iodine salts.

The fortification of pumpkins may also constitute an attractive alternative source of iodine for all consumers, especially vegetarians and vegans [16,17,42]. However, the suitability of this type of product as a matrix for food enrichment may be determined by factors influencing the stability of iodine [43,44,45]. The complexity of iodine fortification encompasses the choice of the chemical form of the compound and the optimum time and hydration [28] of the matrix. For the iodine fortification process to be as effective as possible, the conditions of impregnation process must be carefully selected, particularly relating to the degree of hydration and the temperature of the impregnated samples before drying. As the most effective parameters for fortifying the dried products, it is recommended to impregnate the vegetables with hydration of 1:1 or 1:2 and an impregnation temperature of −76 °C [28].

Preliminary studies support the possibility of using pumpkin as an iodine matrix. However, the difficulty may lie in selecting a pumpkin variety to preserve the highest iodine stability during production and storage. Additionally, iodine-enriched food should be easily and quickly produced, and it should improve rather than lower the health-promoting properties of fortified food [46]. High doses of iodine (up to 3 mg/100 g) are often used in the preparation of iodine-fortified concentrates [47]. Preliminary studies indicated the existence of certain correlations between the antioxidant activity and content of iodine.

It was found that both KI and KIO_3_ have different pro- and antioxidative properties; KI is the reductant, whereas KIO_3_ is the oxidant and may react with oxidizable substances [48].

It was confirmed that potassium iodide only increased lipid peroxidation when used in the highest concentrations (≥50 mM), whereas potassium iodate increased lipid peroxidation in concentrations from 2.5 mM [49]. Many studies confirmed the antioxidant properties of pumpkin [17,50,51,52]. However, the antioxidant activity of pumpkins fortified with iodine was not tested. Therefore, this study aimed to investigate the use of selected pumpkin varieties as carriers of potassium iodide (KI) and potassium iodate (KIO_3_) at different concentrations. It was hypothesized that the different pumpkin varieties affect iodine stability during the storage of enriched dried product and its antioxidant activity. It was also hypothesized that the concentrations of pumpkin iodine fortification would affect the antioxidant activity of fortified pumpkin.

## 2. Materials and Methods

### 2.1. Material

Pumpkin varieties, i.e., *Cucurbita pepo* (Spaghetti (Sp) and Delicata (Dl)) and *Cucurbita moschata* (Butternut Squash (BtnS), Butterkin (Btk), Shishigatani (Ss), Butternut Orange (BtnOr), and Muscat Provence (MsP)), were used as a matrix for the iodine. The plant material was from farms and marketplaces in the Wielkopolska region of Poland. Purchases were made during the harvest of pumpkin, in September–October (2021), when they reached maturity. The pumpkin was then transported to the laboratory and prepared for further analysis.

All pumpkin varieties contained iodine in the amount below 0.001 mg/100 g. The products were purchased in retail trade. KI and KIO_3_ constituted the sources of iodine (Merck, Darmstadt, Germany). 

The DPPH^•^-scavenging capacity was tested using the DPPH radical (2,2-difenylo-1-pikrylhydrazyl) (Sigma-Aldrich, Saint Louis, MO, USA). The ABTS^•+^-scavenging capability was tested using the ABTS radical cation (2,2′-azino-bis(3-ethylbenzothiazoline-6-sulfonic acid) diammonium salt) (98%), (Sigma-Aldrich, Saint Louis, MO, USA). 

The DPPH- and ABTS-scavenging capability was tested using Trolox (6-hydroxy-2,5,7,8-tetramethylchromane-2-carboxylic acid) (97%) (Sigma-Aldrich, Saint Louis, MO, USA).

#### 2.1.1. Conditions of Impregnation

The pumpkins were washed under running tap water and peeled with knives, and the pumpkin seeds were removed. 

All the pumpkin samples were cut into small pieces of approximately 4 × 4 × 4 cm. Next, the samples were steamed (100 °C; 10 min) in a convection oven (Rational, Landsberg am Lech, Germany). The pumpkin samples were subsequently drained and subjected to homogenization (homogenizer—Foss, Hilleroed, Denmark). The next impregnation stage was the soaking of the pumpkin samples in the aqueous solution of KI/KIO_3_. 

In the research, the model adopted three variable iodine concentrations: 0.023 mg/kg (0.030 mg/100 g of KI or 0.039 mg/100 g of KIO_3_—low iodine level in food products), 0.23 mg/kg (0.30 mg/100 g of KI or 0.39 mg/100 g of KIO_3_—natural iodine levels in foods from iodized salt), and 2.3 mg/100 g (3.01 mg/100 g of KI or 3.88 mg/100 g of KIO_3_—fortified matrices for food fortification).

The following conditions of impregnation were assumed: the degree of hydration in the ratio 1:1 (m/v) and incubation at −76 °C/12 h. Then, the impregnated preparations were freeze-dried to the moisture content at the level of 4–5%. The dried pumpkin samples were subjected to homogenization (homogenizer—Foss, Hilleroed, Denmark) to obtain a powder particle size of approximately 250 μm (fine sieves—Sigma-Aldrich, Taufkirchen, Germany). 

#### 2.1.2. Storage Conditions of Iodine Sources

The impregnated and freeze-dried samples of pumpkin under investigation were stored in jars (black glass, closed with a screw top, d = 7 cm, h = 10 cm). The influence of storage conditions on the stability of KI and KIO_3_ was tested during storage of 21 ± 1 °C. Our research confirmed that this temperature is often used and favorable for storing dried vegetables, wheat dietary fiber, and soy protein [26,53]. The iodine contents in the investigated carriers were monitored for 320 days.

### 2.2. Methods

#### 2.2.1. Stability of Iodine

To determine the effectiveness of the iodine impregnation conditions, the iodine content of the pumpkin samples was determined after the application of iodine and storage. The iodine contents in the investigated carriers were monitored on selected storage days: 1, 60, 120, 180, 240, and 320.

The quantitative changes in the total iodine were determined using a macro chemical method with potassium thiocyanate described by Kuhne, Wirth, and Wagner [54], as well as subsequent colorimetric analysis, according to the method described by Moxon and Dixon [55].

#### 2.2.2. Antioxidant Activity

All pumpkin samples, after drying and 320 days of storage, were taken for analysis of antioxidant activity. Ethanol extracts of pumpkin were prepared by 2 h maceration of dried pumpkin (10 g) with 100 mL of 80% ethanol [50]. 

The antioxidant activity of the tested ethanol extracts of pumpkins with iodine was examined on the basis of the free-radical scavenging indices—the DPPH-scavenging capacity (DPPH^•)^ and the ABTS-scavenging capability (ABTS^•+^).

The DPPH^•^-scavenging capacity [56,57] was tested using spectrophotometric methods with the use of DPPH radical. The resultant mixture was shaken thoroughly and allowed to stand at room temperature in the dark for 30 min, after which the absorbance of the solution was measured at 517 nm. The result was expressed as mg Trolox/100 g dry matter of extract.

The ABTS^•+^-scavenging capability [58] was tested using spectrophotometric measurement of changes in the concentration of ABTS radical cation (98%). The absorbance was measured at 734 nm. The DPPH- and ABTS-scavenging capability was tested with regard to the scavenging capacity of Trolox (97%). The result was expressed as mg Trolox/100 g dry matter of extract.

### 2.3. Statistical Analysis

The obtained results were subject to statistical analysis using the STATISTICA TM PL 13.3 (StatSoft, Cracow, Poland) software. The software was used to calculate significant differences between means (*p* < 0.05; analysis of variance ANOVA and Tukey’s multiple range test).

The iodine content and the antioxidant activity of the tested samples were analyzed in six samples (two independent samples and three measurements for each sample). Hypotheses were tested at α = 0.05. To predict the dynamics of changes in iodine content in carriers during the storage, losses of 25% (T _25%_) were used. This term describes the time within which the initial iodine content decreased by 25%. This parameter was calculated from an exponential decay mode [53]. The accuracy of the models was estimated using the coefficient of determination (R^2^) and root-mean-square error (RMSE). The significance level for all analyses was set at 5%.

## 3. Results

### 3.1. Iodine Stability

#### 3.1.1. Iodine Stability after Drying Pumpkins Fortified with Iodine

It was found that all samples of pumpkin proved to be a good material for fortification with iodine. Table 1 shows the iodine content (%) of enriched pumpkin varieties after the drying process of samples fortified with iodine KI and KIO_3_. The differences in iodine content in the range of 85–95% were confirmed, depending on the form of iodine, variety of pumpkin, and concentration of iodine. The analysis of variance (one-way ANOVA test) showed (Table 2) a statistically significant effect (*p* < 0.05) of the type of iodine compound used for fortification (KI, KIO_3_). Both iodine forms showed a capacity to accumulate iodine in large amounts, with a higher iodine concentration noted for KIO_3_ than KI. The analysis of variance (one-way ANOVA test) also showed a statistically significant effect (*p* < 0.05) of the pumpkin varieties. The accumulation of iodine in fortified pumpkin depended on the amount of iodine applied. However, the amount of iodine applied did not affect the percentage of the recovery of the analyzed component. Taking all the predictive factors into account (Table 2), the iodine form was confirmed to have a stronger effect on the final iodine content than the varieties of pumpkin and iodine concentration. The lowest iodine content (85%) was found in KI-fortified *C. moschata* Butterkin (Btk) samples. The highest reproducibility of iodine was found when the pumpkin matrix was fortified using KIO_3_ for *C. moschata* Delicata (Dl) varieties.

#### 3.1.2. Iodine Stability during Storage of Dried Pumpkins Fortified with Iodine

The experiment assumed storage of dried all varieties pumpkin at 21 °C. The tables containing all the iodine concentration data are included in Supplementary Tables S1 and S2. 

The statistical analysis (one-way ANOVA test) (Table 3) showed the strongest correlation between the forms of iodine (KI/KIO_3_) (F = 4239.00; *p* < 0.05) and iodine stability during storage.

**Figure 1:**
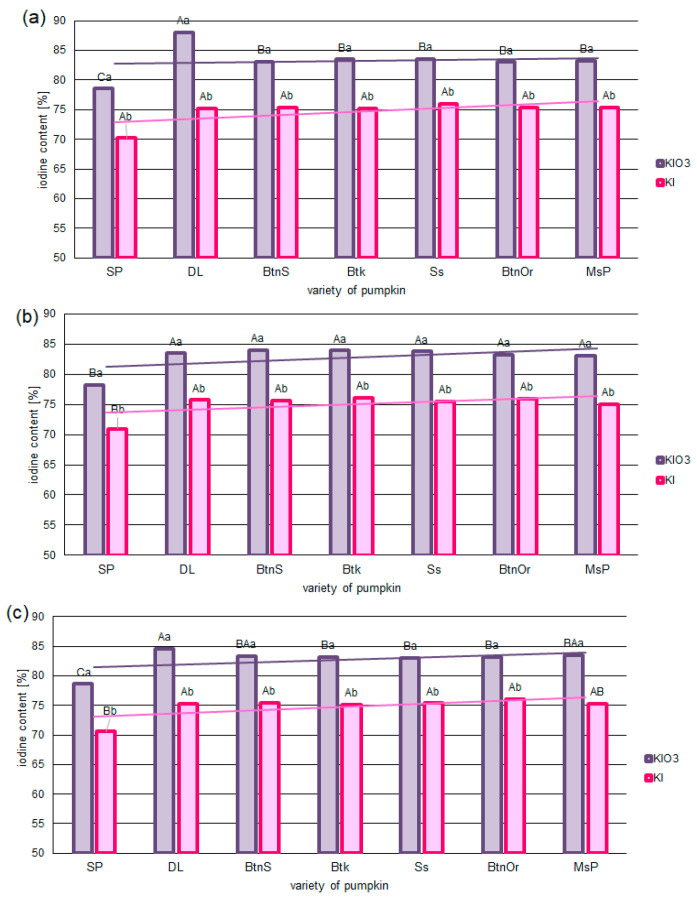
Iodine content [%] in selected varieties of pumpkin fortified with KIO_3_ or KI in concentrations of 0.023 mg/100 g (**a**), 0.23 mg/100 g (**b**), and 2.3 mg/100 g (**c**). Mean values (n = 6); different letters (lower letters in the same varieties of pumpkin; upper case letters in the same form of iodine) denote a significant difference at *p* < 0.05 (one-way ANOVA and post hoc Tukey test).

The iodine content of fortified pumpkins after 320 days of storage differed, ranging from 83% to 70% of the initial content after drying. Iodine losses were lower for samples impregnated with KIO_3_ than KI. This was true for all concentrations of iodine and pumpkin varieties applied. The exception to this was the sample of the Spaghetti (Sp) variety. This sample had the lowest content of KIO_3_ and KI after 320 days of storage (Figure 1a–c). The highest iodine content was found when the pumpkin matrix was fortified using KIO_3_ for the Delicata (Dl) variety (83–88%).

Analysis of the dynamics of changes in iodine content (T _25%_) (Table 4) confirmed a faster rate for KI than for KIO_3_. Similar dynamics of iodine loss during storage for all pumpkin varieties were found. A statistically significantly faster rate of iodine loss was found only for spaghetti pumpkin. For these varieties, the rate of iodine loss was faster by 10% for KI and 8% for KIO_3_.

### 3.2. Antioxidant Activity of Dried Pumpkins Fortified with Iodine after Drying and 320 Days of Storage

The antioxidant activity of the tested ethanol extracts of all pumpkin varieties was examined on the basis of free-radical scavenging indices—the ABTS-scavenging capability (DPPH^•^) and the DPPH-scavenging capacity (ABTS^•+^). The analysis concerned samples after drying and after storing for 320 days. The results of our study confirmed the antiradical effect of all pumpkin varieties on DPPH^•^ and ABTS^•+^. 

#### 3.2.1. ABTS Radical-Scavenging and DPPH Radical-Scavenging Activity in the Samples without Iodine Fortification 

The ABTS^•+^ test results showed that the variety of Delicata exhibited the highest antioxidant activity (149.30 mg Trolox/100 g dm) (Table S3). Strong antioxidant activity was also found for the Spaghetti variety. A high ability to scavenge cation radicals was also found among all varieties of *C. moschata* pumpkins. The highest ABTS^•+^ was confirmed in varieties of Butterkin and Shishigatani. Similar activity was confirmed for other pumpkin varieties. The lowest ability to neutralize ABTS^•+^ was found in the variety of Muscat Provence (99.36 mg Trolox/100 g dm).

Similarly to the previous results, a high ability to scavenge free radicals in the DPPH^•^ test was found for all pumpkin varieties. The highest antioxidant activity was observed in *C. pepo* cultivar Delicata (155.50 mg Trolox/100 g dm). In the case of *C. moschata*, the highest antioxidant potential was confirmed for varieties of Butternut Orange, Shishigatani, and Butternut Squash (Table S4). The lowest ability to neutralize DPPH^•^ was found for the Butterkin (69.78 mg Trolox/100 g dm).

#### 3.2.2. ABTS Radical-Scavenging and DPPH Radical-Scavenging Activity in the Samples with Iodine Fortification 

The statistical analysis (Table 5) confirmed a relationship between the forms of iodine (KI/KIO_3_) and the ABTS^•+^ and the DPPH^•^ test results.

The strongest relationship (one-way ANOVA test) was confirmed between iodine concentration in the form KIO_3_ and the ABTS^•+^ (F = 3836.00; *p* < 0.05) and the DPPH^•^ test results (F = 902.00; *p* < 0.05).

It was observed that, in the systems with iodine concentration at 0.023 and 0.23 mg/100 g (0.030 and 0.301 mg/100 g of KI or 0.039 and 0.388 mg/100 g of KIO_3_), the antioxidant activity based on free-radical scavenging capacity indices (ABTS^•+^ and DPPH^•^) did not change. This was confirmed for the samples after drying and 320 days of storage.

It was also found that fortification with KI concentration at 2.3 mg/100 g caused no significant changes in antioxidant activity in the iodine-fortified pumpkin. A statistically significant decrease in the free-radical scavenging indices was only observed in systems containing varieties of Delicata and Butterkin (with KI at 2.3 mg/100 g). This was confirmed for the samples after drying and 320 days of storage. In the samples containing KI at 2.3 mg/100 g, the capacity to terminate the ABTS^•+^ decreased by 3% compared to samples without iodine. This was confirmed for samples (dried Delicata and Butterkin) after drying and 320 days of storage. The values to neutralize DPPH^•^ decreased by 3% (for samples after drying) and 5% (for samples after 320 days of storage) compared to samples without iodine. These values were confirmed for both pumpkin varieties (Delicata and Butterkin). 

**Figure 2:**
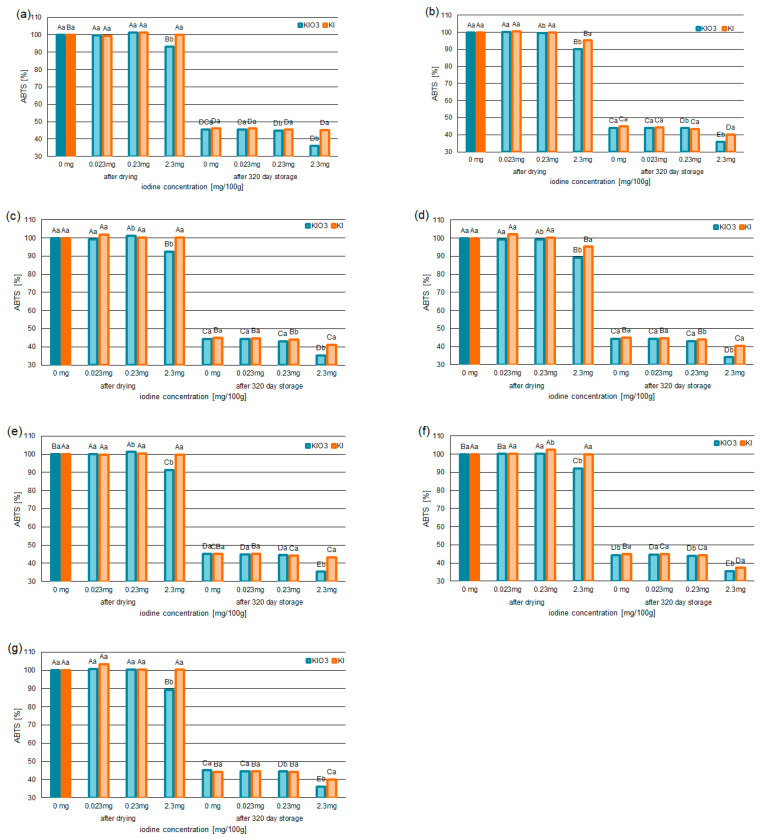
The ABTS^•+^-scavenging capability of selected varieties of pumpkin: Spaghetti (**a**), Delicata (**b**), Butternut Squash (**c**), Butterkin (**d**), Shishigatani (**e**), Butternut Orange (**f**), and Muscat Provence (**g**): fortified with iodine KI and KIO_3_ (concentration: 0.023 mg/100 g, 0.23 mg/100 g, and 2.3 mg/100 g), compared to samples without iodine. Mean values (n = 6); different letters (lower letters in the same varieties of iodine concentration; upper case letters in the same form of iodine) denote a significant difference at *p* < 0.05 (one-way ANOVA and post hoc Tukey test).

**Figure 3:**
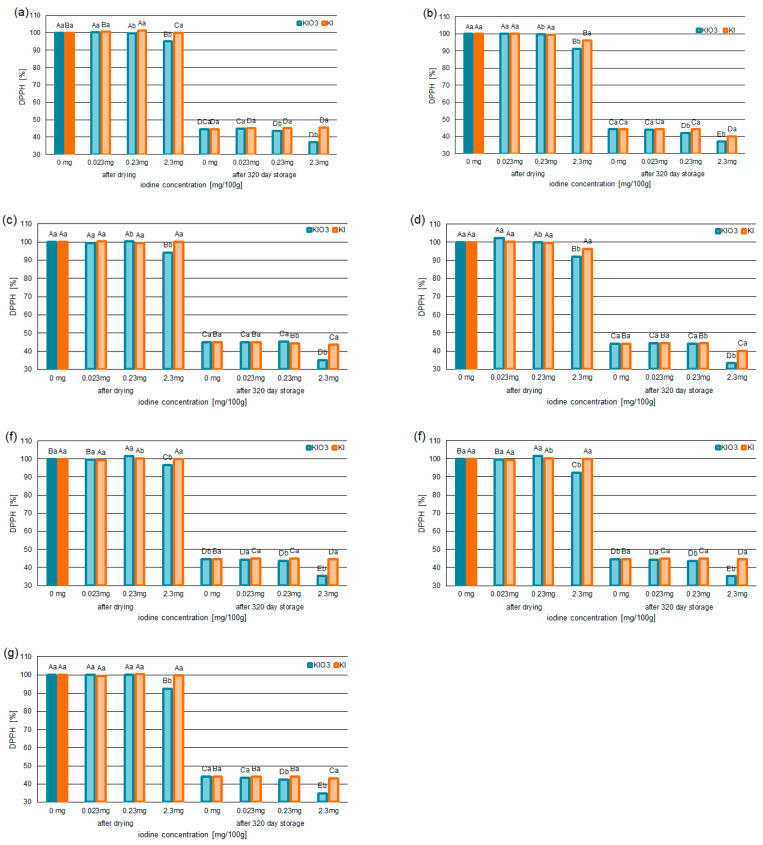
The DPPH^•^-scavenging capability of the selected varieties of pumpkin: Spaghetti (**a**), Delicata (**b**), Butternut Squash (**c**), Butterkin (**d**), Shishigatani (**e**), Butternut Orange (**f**), and Muscat Provence (**g**); fortified with iodine KI and KIO_3_ (in concentration: 0.023 mg/100 g, 0.23 mg/100 g, 2.3 mg/100 g), compared to samples without iodine. Mean values (n = 6); different letters (lower letters in the same varieties of iodine concentration; upper case letters in the same form of iodine) denote a significant difference at *p* < 0.05 (one-way ANOVA and post hoc Tukey test).

For all pumpkin varieties fortified with KIO_3_ concentration at 3.8 mg/100 g (2.3 mg/100 g of iodine), a statistically significant decrease in free-radical scavenging (Figure 2a–g and Figure 3a–g) was confirmed. The capacity for ABTS^•+^ scavenging decreased by 7–11% (for samples after drying) and 7–9% (for samples after 320 days of storage) compared to samples without iodine. The lowest ability to neutralize ABTS^•+^ was found in the iodine-fortified Butterkin variety with 2.3 mg iodine/100 g. This was confirmed in the samples after drying (from 128.36 mg Trolox/100 g to 114.56 mg Trolox/100 g) and 320 days of storage (from 56.77 mg Trolox/100 g to 43.68 mg Trolox/100 g).

Similarly, the deactivation of DPPH^•^ was reduced. In the samples with KIO_3_ at the level of 2.3 mg, the DPPH-scavenging capability was found to be reduced by 6–8% for the samples after drying, while, for the samples after 320 days of storage, the scavenging of DPPH^•^ decreased by 8–11%. The largest reduction in DPPH^•^ was confirmed for the variety of Butterkin.

## 4. Discussion

Approximately 30% of the world’s population remains at risk of iodine deficiency. Therefore, the need to enrich food with iodine justifies research to find a new matrix for iodine fortification.

Analysis of iodine content showed a high recovery of the introduced iodine in all pumpkin varieties after drying to 95%. Previous data on the fortification of protein preparations and vegetables confirm the maximum reproducibility of iodine in fortified matrices at a similar level [28]. Furthermore, high iodine stability in storage was confirmed. The dynamics of changes in iodine content during storage were similar to previous data on the fortification of protein preparations [53] and vegetables [28].

Both iodine forms showed a capacity to accumulate iodine in large amounts, with higher iodine stability noted for KIO_3_ than KI, which was confirmed by earlier studies [53,59,60]. This particularly affected the stored samples. This observation is explained by the lower stability of KI and a higher rate of iodine transformation transition to free iodine [61].

The high stability of KIO_3_ is explained by the mechanism of the iodine form transformation. Iodate is reduced to iodide, and potassium iodide behaves like a simple ionic salt and is easily oxidized to I^2^ [49,62,63].

This increased stability of iodine applied to pumpkins may also be related to the appropriate protein content and lower pH [24,64,65]. This was confirmed by previous data on impregnating vegetables with thiamine and iodine [26,28]. Iodine applied to food remains in the form of inorganic iodine (I^2^), but some of its parts interact with protein or phenolic compounds. Dried pumpkin contains protsein on the level of 10 g, which promotes the formation of complexes with the protein. It was found that the stability of organic iodine in food is higher than that of inorganic iodine [59,66]. Other studies also confirmed that iodine effectively stabilized in the pH range of 1.5–10.5, similar to the pH of pumpkin [62,66]. Other data indicated that pH 8 seemed to have the most adverse effects on iodine stability [62,66]. A study also showed iodine’s instability at pH values < 1.5 and >10.5, and suggested optimal iodine stability (without appreciable iodine volatilization) in the 1.5 ≤ pH < 7 range [67].

The ability to scavenge free radicals is one of the most essential features that determine high antioxidant properties [5,17,50,51,68,69,70]. The results of our study confirmed the antiradical effect of all pumpkin varieties using the DPPH^•^ and ABTS^•+^ methods. Previous studies also indicated a high antioxidant activity of dried pumpkins [52,71]. Moreover, this research showed the highest ability to scavenge cation radicals for pumpkins Delicata, Butterkin and Shishigatani. Other studies also confirmed that pumpkin polysaccharides possess antioxidant activities and can be utilized to develop antioxidant food and medicines [72,73]. 

These trends were also confirmed in the samples during storage. However, a significant reduction was found in the ability to scavenge the free radicals of all pumpkin varieties. An earlier study also confirmed that, after storage, a significant decrease in the antioxidant activity of pumpkins was observed. However, the samples with pumpkin additives still presented a higher score than the control samples [52]. Other results also showed that the antioxidant activities of juice from *Momordica charantia* L. decreased substantially after 3 days of storage. It was found that this antioxidant activity decreased more rapidly at higher storage temperatures [74]. Similar trends were also found for dried kale (*Brassica oleracea* L. var. acephala) [75]. 

Our studies show that, in the systems with iodine concentration at 0.3 and 0.03 of KI and 0.39 and 0.039 mg/kg of KIO_3_, the antioxidant activity based on free-radical scavenging indices (ABTS and DPPH assays) did not change.

Moreover, according to Krzepiłko et al. [76], iodization of radish sprouts did not affect the total antioxidant capacity of hydrophilic antioxidants measured by the ABTS method or hydrophobic antioxidants measured by the DPPH method in either cultivar. In contrast, Blasco et al. [77] showed increased antioxidant activity in lettuce iodized with KI.

Our studies showed that, in fortifying with KIO_3_ at 3.9 mg/100 g (2.3 mg/100 g of iodine), the capacity to scavenge the ABTS^•+^ and DPPH^•^ radicals decreased. This applied to all pumpkin varieties. 

An earlier study confirmed that KI and KIO_3_ have different pro- and antioxidative properties; KI is the reductant, while KIO_3_ is the oxidant [78,79,80]. The research of Iwan et al. [48] showed the prooxidative effects of KIO_3_ when this prooxidant was used in doses resulting in physiological concentrations of iodine in the thyroid. According to Milczarek [49], KIO_3_ increased lipid peroxidation in porcine thyroid homogenates in concentrations ≥ 2.5 mM. The damaging effect of KIO_3_ increased gradually from the concentration of 2.5 mM to 10 mM. The strongest damaging effect was observed at the KIO_3_ concentration of 10 mM. The research of Bürgi et al. confirmed the oxidative potential of iodate [81]. However, it was confirmed that, among three halogenate salts, i.e., iodate, bromate, and chlorate, the first was characterized by the lowest redox potential [49,81]. At the same time, iodine used as KI did not reveal in the present study any toxic effects on membrane lipids, and it even prevented experimentally induced lipid peroxidation when used in the same range of concentrations. This study supported that the use of KI in iodine fortification is safer than KIO_3_, in terms of their influence on oxidative damage to macromolecules [49].

An earlier study also confirmed that iodine, especially iodate, may react with oxidizable substances [82]. Previous research also confirmed the correlation between the content of bioactive compounds and the pumpkin pulp’s antioxidant activity. According of Krzepidło et al. [76], Kulczyński and Gramza [64], and Stryjecka et al. [83], the major antioxidant compounds in plants are phenolic compounds, ascorbic acid, and compounds containing thiol groups. It was confirmed that fortification with high levels of iodine reduced the content of phenolic compounds [76] and may react with oxidizable substances [78]. This decrease in antioxidant activity in the presence of KIO_3_ at high concentrations can also be related to the reactions of iodine with antioxidant proteins [84,85,86]. It was found that a polysaccharide with a low molecular weight (3.5 kDa) extracted from pumpkin displayed antioxidant activities toward free radicals [23]. Other research suggested a relationship among the formation of sulfur compounds (sulfhydryl and disulfide groups), amino acids (cysteine), and high concentrations of iodine [87]. This was confirmed by the better-known substitution reactions of iodine with proteins involving tyrosine or histidine. Similarly, an earlier study confirmed that higher contents of available lysine characterized the meatballs iodized with KI than those iodized with KIO_3_ [88]. A relationship was found among the formation of these complexes, protein function changes, and antioxidant activity. The conclusions of Hassan (2018) suggested a relationship between the antioxidant activity of products with pumpkin and vitamin C content [24]. Other research confirmed a dependence between the degradation of ascorbic acid to dehydroascorbic acid (or reverse) and a high concentration of iodine. Ascorbic acid is a strong reducing agent that quenches any singlet oxygen present, formed during oxidation reactions in foods. Ascorbic acid may react preferentially with iodate, if present, rather than oxygen and, thus, be lost as an antioxidant.

Only a few studies have been performed until now to compare the antioxidant effects of iodine present in two different sources, namely, KI and KIO_3_. Therefore, in future studies, it is worthwhile to further develop this topic by studying, for example, the negative effect of a more varied range of concentrations of iodine and the possibility of the interaction of iodine with other ingredients of vegetables and related oxidative effects. Particular regard should be given to analyzing the relationship between different carbohydrate–protein profiles of the pumpkin fortified with iodine and their antioxidant stability.

## 5. Conclusions

High recovery of the introduced iodine in all investigated pumpkin varieties after drying and high stability during storage make them an attractive source of the matrix for iodine. Pumpkins of all varieties can be fortified with iodine, with higher stability obtained using KIO_3_. However, the research on all pumpkin varieties fortified with KIO_3_ at 3.9 mg/100 g (2.3 mg/100 g of iodine) confirmed a statistically significant decrease in free-radical scavenging. Therefore, for maximum effectiveness in pumpkin’s free-radical scavenging indices, it is suggested to introduce iodine in the form of KI and KIO_3_, but in controlled concentrations. KIO_3_ should be added at a maximum amount of 0.39 mg/100 g.

Therefore, to maintain iodine stability and the high antioxidant activity of pumpkin, it is necessary to consider iodine addition to the systems in concentrations that limit their interactions.

## Figures and Tables

**Table 1:** Iodine content [%] in selected varieties of pumpkin fortified with KIO_3_ and KI.

Iodine Form	Pumpkin Variety	Iodine Concentration [mg·kg^−1^]					
		0.023		0.23		2.30	
		%	Standard Deviation	%	Standard Deviation	%	Standard Deviation
KIO_3_	Sp	93.59 ^aB^ *	0.14	93.75 ^aB^	0.21	93.45 ^aB^	0.12
	Dl	95.45 ^aA^	0.28	95.11 ^aA^	0.25	95.03 ^aA^	0.19
	BtnS	93.42 ^aB^	0.25	93.35 ^aB^	0.39	93.89 ^aB^	0.21
	Btk	93.12 ^aB^	0.19	93.13 ^aB^	0.28	93.23 ^aB^	0.25
	Ss	93.87 ^aB^	0.29	93.38 ^aB^	0.19	93.12 ^aB^	0.19
	BtnOr	92.98 ^aB^	0.21	93.12 ^aB^	0.21	92.89 ^aB^	0.19
	MsP	93.12 ^aB^	0.19	93.45 ^aB^	0.19	93.01 ^aB^	0.26
KI	Sp	87.25 ^aB^	0.19	87.03 ^aB^	0.29	87.98 ^aB^	0.14
	Dl	90.03 ^aA^	0.20	89.45 ^aA^	0.22	89.6 ^aA^	0.12
	BtnS	89.45 ^aB^	0.19	88.38 ^aB^	0.15	88.75 ^aB^	0.23
	Btk	84.78 ^aC^	0.28	85.03 ^aC^	0.19	85.02 ^aC^	0.31
	Ss	87.54 ^aB^	0.11	87.69 ^aB^	0.25	87.99 ^aB^	0.25
	BtnOr	88.21 ^aB^	0.14	88.02 ^aB^	0.28	87.98 ^aB^	0.14
	MsP	87.02 ^aB^	0.21	88.12 ^aB^	0.14	87.42 ^aB^	0.21

* Mean values (n = 6); different letters (lower case letters in the same varieties of pumpkin; upper case letters in the same concentration of iodine) denote a significant difference at *p* < 0.05 (one-way ANOVA and post hoc Tukey test).

**Table 2:** Statistical significance of predictors of variance models for changes in iodine content in selected iodine-fortified pumpkins after drying (one-way ANOVA test).

Predictors	SS	df	MSE	F-Value	*p*-Value
Iodine concentration	0.07	2	0.04	0.09	0.91
Pumpkin type	111.70	6	18.62	48.36	0.00
Iodine form	1075.73	1	1075.73	2794.51	0.00

SS—statistical significance; df—degrees of freedom; MSE—mean sum of squares.

**Table 3:** Statistical significance of predictors of variance models for changes in iodine content in selected iodine-fortified pumpkins after 320 days of storage (one-way ANOVA test).

Predictors	SS	df	MSE	F-Value	*p*-Value
Iodine concentration	1.50	2	0.80	2.00	0.15
Pumpkin type	423.70	6	70.60	180.00	0.00
Iodine form	1667.10	1	1667.10	4239.00	0.00

SS—statistical significance; df—degrees of freedom; MSE—mean sum of squares.

**Table 4:** Dynamics of changes in iodine content (mg·kg^−1^) during 320 days of storage of the dried iodine-fortified pumpkins with various iodine concentrations and pumpkin types.

Parameters Fortifications		Dynamics of Change in Iodine Content over 320 Days									
Pumpkin Variety	Iodine Concentration [mg·kg^−1^]	T_25%_ [days]	*R* ^2^	*RMSE*	k *	A_0_ *	T_25%_ [days]	*R* ^2^	*RMSE*	k *	A_0_ *
	KIO_3_						KI				
Sp	0.023	341.50	0.978	0.000	−0.0000	1.022	255.14	0.989	0.000	0.000	1.020
	0.23	343.24	0.979	0.002	−0.0002	1.239	258.22	0.989	0.001	0.000	1.220
	2.3	343.48	0.987	0.019	−0.0015	8.427	263.22	0.990	0.010	−0.001	7.484
Dl	0.023	517.58	0.948	0.000	−0.0000	1.022	336.73	0.985	0.000	0.000	1.021
	0.23	520.89	0.879	0.002	−0.0002	1.242	338.08	0.982	0.001	0.000	1.228
	2.3	490.05	0.982	0.004	−0.0015	8.864	345.75	0.975	0.006	−0.001	7.806
BtnS	0.023	478.23	0.996	0.000	0.0000	1.022	336.27	0.981	0.000	0.000	1.021
	0.23	484.35	0.996	0.000	−0.0001	1.239	339.41	0.981	0.001	0.000	1.225
	2.3	505.16	0.996	0.003	−0.0005	8.638	342.57	0.984	0.006	−0.001	7.650
Btk	0.023	482.99	0.996	0.000	0.0000	1.022	336.15	0.976	0.000	0.000	1.020
	0.23	476.69	0.996	0.000	−0.0001	1.239	335.30	0.971	0.000	0.000	1.216
	2.3	503.81	0.996	0.004	−0.0007	8.500	343.46	0.975	0.004	−0.001	7.040
Ss	0.023	487.16	0.983	0.000	0.0000	1.022	342.63	0.985	0.000	0.000	1.020
	0.23	473.25	0.980	0.000	0.0000	1.239	339.90	0.981	0.015	0.000	1.223
	2.3	496.96	0.981	0.003	−0.0005	8.490	337.43	0.984	0.006	−0.001	7.520
BtnOr	0.023	473.34	0.986	0.000	0.0000	1.022	335.03	0.982	0.000	0.000	1.020
	0.23	469.47	0.985	0.000	0.0000	1.239	340.52	0.984	0.001	0.000	1.224
	2.3	483.69	0.978	0.003	−0.0004	8.449	339.40	0.987	0.007	−0.001	7.514
MsP	0.023	476.90	0.987	0.029	−0.0000	1.022	337.05	0.986	0.000	0.000	1.020
	0.23	483.44	0.987	0.057	−0.0001	1.243	328.70	0.986	0.006	0.000	1.230
	2.3	482.76	0.985	0.072	−0.0011	8.652	332.40	0.981	0.006	−0.001	7.425

* A_0_—the initial amount of iodine, k—decay constant [59].

**Table 5:** Statistical significance of predictors of covariance models for changes in the ABTS^•+^- and the DPPH^•^-scavenging capacity in the selected iodine-fortified pumpkins after drying and 320 days of storage (one-way ANOVA test).

Predictors	SS	df	MSE	F-Value	*p*-Value
ABTS^•+^					
after drying, fortified with KI0_3_					
Iodine concentration	1352.30	3	450.8	510.00	0.00
Pumpkin type	26.90	6	4.51	5.11	0.00
after drying, fortified with KI					
Iodine concentration	68.50	3	22.80	15.30	0.00
Pumpkin type	38.61	6	6.40	4.30	0.00
after 320 days of storage, fortified with KIO_3_					
Iodine concentration	1277.7	3	425.90	3836.00	0.00
Pumpkin type	18.62	6	3.12	28.00	0.00
after 320 days of storage, fortified with KI					
Iodine concentration	186.10	3	62.00	65.71	0.00
Pumpkin type	63.30	6	10.61	11.21	0.00
DPPH^•^					
after drying, fortified with KIO_3_					
Iodine concentration	149.90	3	50.10	97.10	0.00
Pumpkin type	11.20	6	1.90	4.12	0.00
after drying, fortified with KI					
Iodine concentration	93.20	3	31.10	5.90	0.00
Pumpkin type	150.30	6	25.00	4.80	0.00
after 320 days of storage, fortified with KIO_3_					
Iodine concentration	1101.4	3	367.1	902.50	0.00
Pumpkin type	19.9	6	3.3	8.10	0.00
after 320 days of storage, fortified with KI					
Iodine concentration	38.40	3	12.81	16.42	0.00
Pumpkin type	34.61	6	5.80	7.40	0.00

SS—statistical significance; df—degrees of freedom; MSE—mean sum of squares.

## Data Availability

The data presented in this study are available on request from the corresponding author.

## Supplementary Materials

The following supporting information can be downloaded at https://www.mdpi.com/article/10.3390/foods12142792/s1: Table S1. Iodine content [mg/100 g] in selected varieties of pumpkin: Spaghetti (Sp), Delicata (Dl), Butternut Squash (BtnS), Butterkin (Btk), Shishigatani (Ss), Butternut Orange (BtnO), and Muscat Provence (MsP), fortified with KIO_3_; Table S2. Iodine content [mg/100 g] in selected varieties of pumpkin Spaghetti (Sp), Delicata (Dl), Butternut Squash (BtnS), Butterkin (Btk), Shishigatani (Ss), Butternut Orange (BtnO), and Muscat Provence (MsP), fortified with KI in concentration; Table S3. The ABTS^•+^-scavenging capacity of selected varieties of pumpkin: Spaghetti (Sp), Delicata (Dl), Butternut Squash (BtnS), Butterkin (Btk), Shishigatani (Ss), Butternut Orange (BtnO), and Muscat Provence (MsP), fortified with iodine KI and KIO_3_ (mg Trolox/100 g dm); Table S4. The DPPH^•^-scavenging capacity of selected varieties of pumpkin: Spaghetti (Sp), Delicata (Dl), Butternut Squash (BtnS), Butterkin (Btk), Shishigatani (Ss), Butternut Orange (BtnO), and Muscat Provence (MsP), fortified with iodine KI and KIO_3_ (mg Trolox/100 g dm).
