# Supplementary material for: The Antioxidant Properties of Selected Varieties of Pumpkin Fortified with Iodine in the Form of Potassium Iodide and Potassium Iodate
Source: Foods. 2023 Jul 23;12(14):2792. doi: 10.3390/foods12142792 (PMC10379304; doi:10.3390/foods12142792)
Supplement: Supplementary file 1 [file foods-12-02792-s001.zip › foods-2453427-supplementary.pdf]

Table S1. Iodine content [mg/100g] in selected varieties of pumpkin: Spaghetti (Sp), Delicata (Dl), Butternut Squash (BtnS), Butterkin (Btk), Shishigatani (Ss), Butternut Orange (BtnO) and Muscat Provence (MsP), fortified with KIO_3.

Pumpkin Variation	Iodine Concentration (mg kg^{-1})	Time of storage [days]					
		0	60	120	180	240	320
Sp	0.23	0.022	0.020	0.019	0.018	0.017	0.017
	2.3	0.216	0.206	0.195	0.184	0.175	0.170
	23.00	2.149	2.043	1.944	1.840	1.751	1.681
Dl	0.23	0.022	0.021	0.020	0.019	0.018	0.017
	2.3	0.219	0.206	0.195	0.183	0.173	0.163
	23.00	2.186	2.147	2.109	2.072	2.035	1.999
BtnS	0.23	0.021	0.021	0.021	0.020	0.020	0.019
	2.3	0.215	0.211	0.208	0.204	0.201	0.197
	23.00	2.159	2.124	2.089	2.055	2.021	1.988
Btk	0.23	0.021	0.021	0.021	0.020	0.020	0.020
	2.3	0.214	0.211	0.207	0.204	0.201	0.197
	23.00	2.144	2.101	2.059	2.017	1.977	1.937
Ss	0.23	0.022	0.021	0.021	0.020	0.020	0.020
	2.3	0.215	0.212	0.209	0.206	0.204	0.201
	23.00	2.142	2.110	2.080	2.049	2.019	1.990
BtnOr	0.23	0.021	0.021	0.021	0.020	0.020	0.020
	2.3	0.214	0.211	0.209	0.206	0.203	0.201
	23.00	2.136	2.108	2.081	2.054	2.027	2.000
MsP	0.23	0.021	0.021	0.020	0.020	0.019	0.018
	2.3	0.215	0.212	0.205	0.196	0.190	0.179
	23.00	2.139	2.087	2.047	1.963	1.889	1.778

Table S2. Iodine content [mg/100g] in selected varieties of pumpkin Spaghetti (Sp), Delicata (Dl), Butternut Squash (BtnS), Butterkin (Btk), Shishigatani (Ss), Butternut Orange (BtnO) and Muscat Provence (MsP), fortified with KI in concentration.

Pumpkin Variation	Iodine Concentration (mg kg^{-1})	Time of storage [days]					
		0	60	120	180	240	320
Sp	0.23	0.020	0.019	0.018	0.017	0.017	0.016
	2.3	0.200	0.191	0.183	0.174	0.166	0.159
	23.00	2.024	1.940	1.861	1.784	1.711	1.641
Dl	0.23	0.021	0.020	0.020	0.019	0.019	0.018
	2.3	0.206	0.201	0.195	0.191	0.186	0.181
	23.00	2.061	2.006	1.953	1.902	1.852	1.803
BtnS	0.23	0.021	0.020	0.020	0.019	0.019	0.018
	2.3	0.203	0.198	0.194	0.189	0.185	0.180
	23.00	2.041	1.982	1.925	1.870	1.816	1.764
Btk	0.23	0.019	0.019	0.019	0.019	0.018	0.018
	2.3	0.196	0.192	0.189	0.186	0.183	0.180
	23.00	1.955	1.916	1.877	1.840	1.803	1.766
Ss	0.23	0.020	0.020	0.019	0.019	0.018	0.018
	2.3	0.202	0.196	0.191	0.186	0.181	0.177
	23.00	2.024	1.968	1.913	1.861	1.809	1.759
BtnOr	0.23	0.020	0.020	0.019	0.019	0.018	0.018
	2.3	0.202	0.197	0.191	0.185	0.180	0.175
	23.00	2.024	1.963	1.905	1.849	1.794	1.740
MsP	0.23	0.020	0.019	0.019	0.018	0.018	0.017
	2.3	0.203	0.197	0.191	0.186	0.180	0.151
	23.00	2.011	1.957	1.904	1.853	1.804	1.755

Table S3. The ABTS•+ of selected varieties of pumpkin: Spaghetti (Sp), Delicata (Dl), Butternut Squash (BtnS), Butterkin (Btk), Shishigatani (Ss), Butternut Orange (BtnO) and Muscat Provence (MsP), fortified with iodine KI and KIO_3 (mg Trolox/100 g dm).

		Iodine Concentration (mg kg^{-1})	KIO_3	KI
Sp	after drying	0 .00	122.30	122.30
		0.23	121.91	121.63
		2.30	123.80	123.80
		23.00	114.02	122.34
	after 320 day storage	0 .00	55.72	56.33
		0.23	55.77	56.23
		2.30	54.77	55.77
		23.00	44.02	55.18
Dl	after drying	0 .00	149.30	149.30
		0.23	149.84	150.32
		2.30	148.78	149.14
		23.00	134.55	142.18
	after 320 day storage	0 .00	65.87	67.23
		0.23	65.71	66.04
		2.30	65.87	64.63
		23.00	53.72	60.06
BtnS	after drying	0 .00	119.30	119.30
		0.23	118.41	121.66
		2.30	120.77	119.57
		23.00	110.19	119.72
	after 320 day storage	0 .00	52.77	53.66
		0.23	52.92	53.16
		2.30	51.44	52.47
		23.00	42.05	48.90
Btk	after drying	0 .00	128.36	128.36
		0.23	127.40	130.90
		2.30	127.40	128.66
		23.00	114.56	122.19
	after 320 day storage	0 .00	56.77	57.74
		0.23	56.94	57.20
		2.30	55.35	56.45
		23.00	43.68	51.74
Ss	after drying	0 .00	132.24	132.24
		0.23	132.28	132.09
		2.30	133.87	132.84
		23.00	120.64	131.95
	after 320 day storage	0 .00	59.81	59.55
		0.23	59.10	59.67
		2.30	58.70	58.23
		23.00	46.63	57.02
BtnOr	after drying	0 .00	109.25	109.25
		0.23	109.27	109.52
		2.30	109.57	111.82
		23.00	100.49	109.25
	after 320 day storage	0 .00	48.46	49.02
		0.23	48.68	49.13
		2.30	48.05	48.34
		23.00	38.74	40.78
MsP	after drying	0 .00	99.36	99.36
		0.23	100.05	102.59
		2.30	99.72	99.68
		23.00	90.40	99.61
	after 320 day storage	0 .00	44.69	43.95
		0.23	44.24	44.08
		2.30	44.11	43.84

		23.00	35.80	39.72

Tables S4. The $DPPH^{\bullet}$ of selected varieties of pumpkin: Spaghetti (Sp), Delicata (Dl), Butternut Squash (BtnS), Butterkin (Btk), Shishigatani (Ss), Butternut Orange (BtnO) and Muscat Provence (MsP), fortified with iodine KI and KIO_3 (mg Trolox/100 g dm).

		Iodine Concentration (mg kg^{-1})	KIO_3	KI
Sp	after drying	0 .00	129.36	129.36
		0.23	129.62	130.03
		2.30	128.83	130.98
		23.00	122.87	129.33
	after 320 day storage	0 .00	57.63	57.63
		0.23	58.19	58.37
		2.30	56.39	58.25
		23.00	48.13	58.87
Dl	after drying	0 .00	155.11	155.11
		0.23	155.50	155.31
		2.30	154.94	154.55
		23.00	141.51	149.26
	after 320 day storage	0 .00	68.61	68.61
		0.23	68.29	68.70
		2.30	65.16	68.61
		23.00	57.75	62.09
BtnS	after drying	0 .00	146.32	146.32
		0.23	145.52	146.66
		2.30	146.66	145.68
		23.00	137.72	146.29
	after 320 day storage	0 .00	65.89	65.89
		0.23	66.02	65.89
		2.30	66.27	64.81
		23.00	51.40	63.74
Btk	after drying	0 .00	69.78	69.78
		0.23	71.34	69.94
		2.30	69.80	69.63
		23.00	64.18	67.21
	after 320 day storage	0 .00	30.79	30.79
		0.23	30.91	31.02
		2.30	30.72	30.85
		23.00	23.23	27.93
Ss	after drying	0 .00	147.25	147.25
		0.23	150.53	147.59
		2.30	147.29	146.93
		23.00	135.65	147.26
	after 320 day storage	0 .00	66.60	66.60
		0.23	65.81	66.44
		2.30	65.36	64.83
		23.00	53.00	66.23
BtnOr	after drying	0 .00	150.00	150.00
		0.23	149.34	149.03
		2.30	152.34	150.35
		23.00	138.47	150.02
	after 320 day storage	0 .00	67.01	67.01
		0.23	66.38	67.55
		2.30	65.51	67.47
		23.00	52.80	66.77
MsP	after drying	0 .00	92.36	92.36
		0.23	92.39	91.77
		2.30	92.39	92.59
		23.00	85.29	91.95

	after 320 day storage	0 .00	40.62	40.62
		0.23	40.23	40.65
		2.30	39.12	40.66
		23.00	32.22	39.91
